# Pharmacological PINK1 activation ameliorates Pathology in Parkinson’s Disease models

**DOI:** 10.21203/rs.3.rs-4356493/v1

**Published:** 2024-05-10

**Authors:** Nicholas Hertz, Randall Chin, Rishi Rakhit, Dara Ditsworth, Chengzhong Wang, Johan Bartholomeus, Song Liu, Akash Mody, Alex Laihsu, Andrea Eastes, Chao Tai, Roy Kim, Jessica Li, Saurabh Khasnavis, Victoria Rafalski, Donald Heerendeen, Virginia Garda, Jennie Phung, Daniel de Roulet, Alban Ordureau, J. Wade Harper, Shawn Johnstone, Jan Stöhr

**Affiliations:** Mitokinin Inc.; Mitokinin Inc.; Mitokinin Inc.; Mitokinin Inc.; Gladstone Institutes/UCSF; X-Chem Inc.; Mitokinin Inc.; Mitokinin Inc.; Mitokinin Inc.; Mitokinin Inc.; Mitokinin Inc.; Mitokinin Inc.; Mitokinin Inc.; AbbVie Inc.; Gladstone Institutes; Mitokinin Inc.; Mitokinin Inc.; Mitokinin Inc.; Mitokinin Inc.; Memorial Sloan Kettering; Harvard Medical School; X-Chem Inc.; AbbVie Inc.

## Abstract

PINK1 loss-of-function mutations and exposure to mitochondrial toxins are causative for Parkinson’s disease (PD) and Parkinsonism, respectively. We demonstrate that pathological α-synuclein deposition, the hallmark pathology of idiopathic PD, induces mitochondrial dysfunction, and impairs mitophagy as evidenced by the accumulation of the PINK1 substrate pS65-Ubiquitin (pUb). We discovered MTK458, a brain penetrant small molecule that binds to PINK1 and stabilizes its active complex, resulting in increased rates of mitophagy. Treatment with MTK458 mediates clearance of accumulated pUb and α-synuclein pathology in α-synuclein pathology models in vitro and in vivo. Our findings from preclinical PD models suggest that pharmacological activation of PINK1 warrants further clinical evaluation as a therapeutic strategy for disease modification in PD.

## Introduction

Parkinson’s Disease (PD) is the second most common neurodegenerative disease, with more than 10 million patients affected worldwide (Ball et al., 2019) and no FDA approved disease modifying therapies (Bloem et al., 2021; Kim et al., 2018; Yang et al., 2020). Hallmark pathologies of idiopathic PD include the formation of Lewy bodies consisting primarily of aggregated α-synuclein and ubiquitin, neuronal loss in the substantia nigra, striatal dopamine deficiency, and mitochondrial deficits (Poewe et al., 2017). These neuropathologic features result in motor deficits such as tremors, bradykinesia, stiffness, and impaired balance, as well as non-motor symptoms like cognitive impairment. The standard of care treatment for PD consists of dopamine replacement therapeutics (*e.g.*, Levodopa), which over time loses clinical efficacy and can cause dyskinesia and dystonia when dose levels increase (Fox et al., 2018; Pringsheim et al., 2021), no effect on the underlying pathologic progression of the disease. Given the absence of disease modifying therapeutic options for patients there is an urgent clinical need to discover and develop therapeutics addressing the underlying pathobiology of PD (Lang et al., 2022; Pagano et al., 2022).

Recent advances in the understanding of the genetic architecture of PD (Nalls et al., 2019) leading to disease initiation and/or affecting disease progression are helping to inform a new generation of therapeutically relevant pathways. Various familial forms of PD have been identified and implicate α-synuclein aggregation, lysosomal dysfunction, and notably, impairments in mitochondrial quality control mechanisms as causative for Parkinson’s disease (Borsche et al., 2021). Homozygous recessive mutations in Parkin, a mitochondrial associated E3-Ubiquitin ligase, or in PINK1, a serine/threonine kinase that serves as a master regulator of mitochondrial quality control, cause DA neuron degeneration and early-onset PD (Kitada et al., 1998; Valente et al., 2004)

Beyond patient genetics, multiple lines of experimental evidence suggest that PD is intimately linked to mitochondrial function, so it has been proposed that improving mitochondrial quality control and homeostasis could be a viable approach for disease modification in PD. It is well established that nigrostriatal dopaminergic (DA) neurons, which degenerate in PD, are particularly susceptible to mitochondrial dysfunction (Fang et al., 2019; Henchcliffe and Beal, 2008). Multiple mitochondrial toxins, including MPTP, rotenone, and paraquat, are linked to Parkinsonism in humans and have been shown to drive preferential degeneration of dopaminergic neurons in in vivo models of PD (Nandipati and Litvan, 2016). Additionally, α-synuclein aggregates have been shown to accumulate at mitochondria, interfere with mitochondrial function, and delay mitophagy (Devi et al., 2008; Ryan et al., 2018; Shaltouki et al., 2018; Wang et al., 2019). Intriguingly, an electron microscopy study of PD patients’ brains identified mitochondrial components within Lewy Bodies (Shahmoradian et al., 2019), further highlighting a potential direct pathogenic interaction of mitochondria-and aggregated α-synuclein. Despite extensive evidence, the hypothesis that ameliorating mitochondrial function may be beneficial for PD has not been thoroughly tested in humans; the clinical trials run to date have focused on using antioxidants to prevent damage rather than augmenting mitochondrial quality control pathways to address the underlying dysfunction (Borsche et al., 2021; Kim et al., 2021). In this work, we explore the idea that clearance of damaged mitochondria and restoring mitochondrial function by activating PINK1 may be beneficial for PD.

Depolarization-induced mitochondrial *auto*phagy, referred to as mitophagy, is tightly regulated by PINK1/Parkin pathway activation. Under basal conditions, PINK1 is targeted to the mitochondrion, cleaved by PARL, and exported for degradation by the proteasome, preventing mitochondrion-localized kinase activity. (Deas et al., 2011; Greene et al., 2012; Jin et al., 2010; Lazarou et al., 2012; Yamano and Youle, 2013). Conversely, in cells undergoing mitochondrial stress, the full length 63 kDa PINK1 is stabilized on the outer mitochondrial membrane and becomes catalytically active. Active PINK1 forms a high molecular weight complex with TOM where it dimerizes and autophosphorylates at Serine 228, potentiating PINK1 activation (Kakade et al., 2022; Lazarou et al., 2013; Okatsu et al., 2012; Ordureau et al., 2014). Active PINK1 directly phosphorylates ubiquitin (Ub) and the ubiquitin-like domain of Parkin, at the homologous Serine residue (Kane et al., 2014; Kazlauskaite et al., 2014; Kondapalli et al., 2012; Koyano et al., 2014; Ordureau et al., 2014; Wauer et al., 2015). Both PINK1-mediated phosphorylation of Parkin and binding to pUb drive activation of Parkin, ultimately leading to the recruitment of additional Parkin to the mitochondrion. Parkin ubiquitinates outer mitochondrial membrane proteins such as the mitofusins (Antico et al., 2021; Bingol et al., 2014; Ordureau et al., 2020; Sarraf et al., 2013), leading to their degradation and causing fragmentation of the stressed parts of the mitochondrial network. The damaged and fragmented pieces of mitochondria are subsequently engulfed by the autophagosome in the process of mitophagy.

Since mitochondrial dysfunction is a key element of PD and loss-of-function mutations in PINK1 result in PD, we sought target PINK1 by developing small molecule activators as a treatment for PD. Building upon our previous discovery of a novel role for kinetin as an activator of PINK1 (Hertz et al., 2013), we synthesized and tested kinetin analogs seeking compounds with superior pharmacological properties leading to the discovery of MTK458. To further understand the role of mitochondrial dysfunction in PD, we found that cells or mice challenged with α-synuclein aggregation exhibit increased pUb, mitochondrial dysfunction, and stalled mitophagy. Remarkably, MTK458 dosing can both, alleviate mitochondrial stress, as evidenced by a decrease in brain and plasma pUb, and decreases the levels of α-synuclein (α-syn) aggregation. These data suggest that pharmacological activation of PINK1 represent an attractive therapeutic strategy by addressing the pathological hallmarks of PD.

## Results

### Pre-formed fibrils of α-synuclein cause mitochondrial dysfunction and impair mitophagy

**Figure 1:**
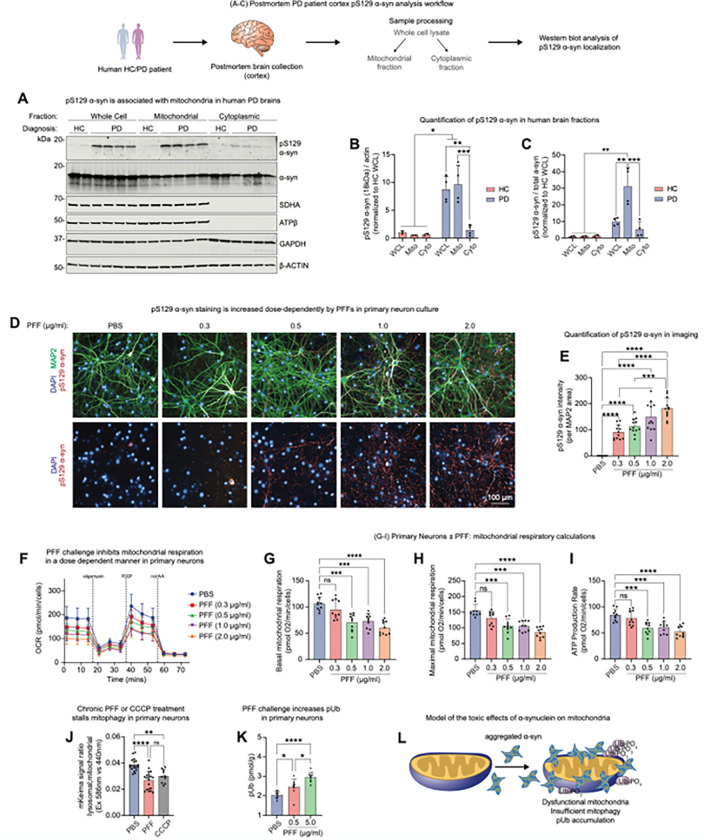
Mitochondria-associated α-synuclein is present in PD patient brain tissue and induces mitochondrial dysfunction in primary neurons. (A) Human brain pieces from HC or PD individuals were separated into mitochondrial or cytoplasmic fractions, or left unfractionated as whole cell lysate, and analyzed by immunoblotting. pS129 α-syn is enriched in the mitochondrial fraction. (B-C) Quantification of (A) is shown. (D-J) M83 primary neuron cultures were challenged with various concentrations of PFFs, and after 21 days, pS129 α-synuclein staining (D-E), mitochondrial respiration (F-I), and mitophagy (J) were assessed. (D-E) pS129 α-synuclein intensity increases dose dependently with PFF challenge. (F-I) PFF challenge dose-dependently decreases basal respiration, maximal respiration, and ATP production rate. (J) Primary neurons challenged for 21 days with 1 μg/ml PFFs or 10 nM CCCP show impaired mitophagy as measured by the mKeima reporter. (K) Primary neurons challenged with PFFs show an accumulation in pUb as measured by the MSD pUb assay. (L) A model of our hypothesis: aggregation of pS129 α-syn occurs at the mitochondria, leading to dysfunctional mitochondria, insufficient mitophagy, and accumulation of pUb. Where applicable, mean and SD are shown; one-way ANOVA was used for statistical analysis. *p < 0.05, **p < 0.01, ***p < 0.001, ****p < 0.0001,n.s., not significant.

The pathological hallmark of idiopathic and most genetic forms of PD is the misfolding and subsequent deposition of α-synuclein into insoluble, beta sheet rich aggregates deposits referred to as Lewy pathology, resulting in the accumulation of posttranslationally modified α-synuclein. One of the most prominent modifications is the phosphorylation at serine 129 (pS129) (Rocha et al., 2018) which is also widely used to detect disease associated α-syn by immunohistochemical and biochemical methods. High-resolution studies revealed that mitochondrial fragments are an integral part of Lewy Body structures, suggesting an interaction of misfolded α-synuclein with mitochondrial structures (Shahmoradian et al., 2019). Furthermore, patient-derived A53T SNCA iPSCs show delayed mitophagy (Devi et al., 2008; Ryan et al., 2018; Wang et al., 2019). Consistent with these findings, we found pS129 α-synuclein associated nearly exclusively with the mitochondrial fraction of cortical brain extracts in human PD patients (Fig. 1A–C). To assess whether pathogenic α-synuclein induced mitochondrial dysfunction we seeded cultured primary neurons with α-synuclein preformed fibrils (PFFs) (Volpicelli-Daley et al., 2011), which lead to pS129 positive α-synuclein accumulation (Figs. 1D–E and S1A-B). We observed concentration- and time-dependent defects in mitochondrial respiration (Figs. 1F–I and S1C-F), impaired mitophagy (Figs. 1J), and a dose-dependent accumulation of pUb (Fig. 1K). Interestingly, a chronic, low dose of the mitochondrial uncoupler CCCP also led to impaired mitophagy in neurons (Fig. 1J) in contrast to the widely used high doses which increase mitophagy (CITATIION). This result suggests that stimulating chronic low levels of mitophagy via mitochondrial uncoupling leads to stalled mitophagy in which PINK1 is partially activated but unable to complete the mitophagic process; as a result, pUb that would otherwise be turned over when the mitophagic process is completed, builds up in the cell. These data support a model in which α-synuclein pathology increases mitochondrial dysfunction and impairs mitophagy, leading to accumulation of pUb (Fig. 1L).

The static observation that pUb is elevated in PFF challenged neurons could indicate reduced or increased PINK1 function, resulting in a decreased or increased rate of mitophagy respectively. This is an often-observed conundrum in autophagy-related processes when measuring adaptor proteins at single time points (CITATION). Parallel measurements of decreased mitophagy levels in this model clearly show that the pUb accumulation is a result of stalled mitophagy, and that increased mitochondrial dysfunction and impaired mitophagy is a caused of α-synuclein aggregation (Figs. 1L), leading to an increase in pUb. Conversely, alleviation of mitochondrial stress and stalled mitophagy by further activation of PINK1 would result in lower pUb and small molecule activators of PINK1 could provide a strategy to resolve stalled mitophagy induced by aggregated α-synuclein restoring the neuronal homeostasis.

### Identifying and qualifying small molecule activators of PINK1

**Figure 2:**
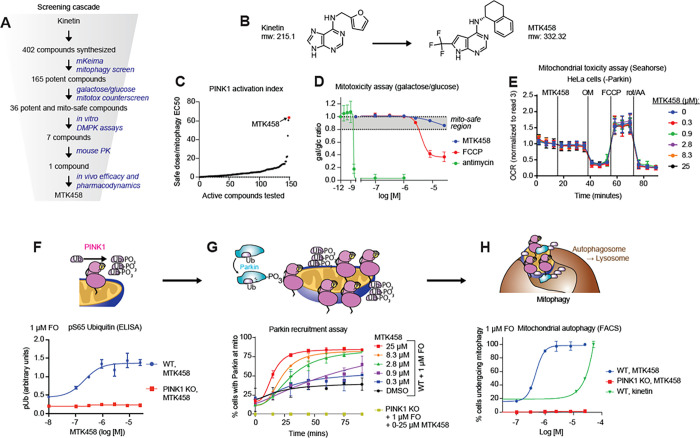
Identification of the small molecule PINK1 activator MTK458 (A) Schematic of the screening funnel used to discover MTK458. (B) The structures of kinetin and MTK458 are shown. (C) Compounds were screened for their ability to induce mitophagy in the mKeima assay. MTK458 is one of the best compounds in terms of potency. (D) SK-OV-3 cells grown in galactose or glucose media were treated with MTK458, FCCP, or antimycin. After 24 hours, the ratio of cells remaining in galactose or glucose media (gal/glc ratio) was plotted. A 20% decrease in the gal/glc ratio was designated as the threshold for a mitotoxic compound. FCCP and antimycin are mitotoxic, but MTK458 is “mito-safe.” (E) HeLa cells (which do not express Parkin) were treated with MTK458 and mitochondrial respiration was assessed by the Seahorse Bioanalyzer. MTK458 does not impair mitochondrial respiration in these cells. (F) YPMK (WT or PINK1 KO) cells were treated with a dose range of MTK458 and 1 μM FCCP/oligomycin (FO), and lysed after 2h. pUb levels were measured by ELISA and shown as raw absorbance units. (G) YPMK (WT or PINK1 KO) cells were treated with MTK458 and 1 μM FO, immediately followed by live cell imaging. The percentage of cells with colocalization of YFP-Parkin with mitochondrial mKeima was assessed at each timepoint. (H) YPMK (WT or PINK1 KO) cells were treated with MTK458 (or kinetin) and 1 μM FO for 6h and then analyzed by FACS to measure the percent of cells undergoing mitophagy. Mean ± SD. *p < 0.05, **p < 0.01, ***p < 0.001, n.s., not significant.

Our experiments establish that reduced rates of mitophagy and unresolved mitochondrial damage are key impairments in α-synuclein pathobiology and support pharmacological activation of PINK1 as a potential avenue to mitigate α-syn-induced cellular impairments. Our initial approach to activate PINK1 with neo-substrates (Hertz et al., 2013) led us to the discovery of kinetin, which activates PINK1 in cells and relieves mitochondrial mutations in flies and mice in a PINK1-dependent manner (Osgerby et al., 2017; Tsai et al., 2022). However, because kinetin has low potency and poor pharmacokinetics (PK) and brain penetrance, we could not detect an effect in PD models in vivo (Orr et al., 2017). To overcome these limitations and to discover novel small molecule PINK1 activators with drug-like properties, we synthesized and screened small molecules derived from the structural core of kinetin (Fig. 2A–B). First, active compounds were discovered by measuring activity in a cell-based assay for mitophagy in which a pH sensitive protein (keima) is localized on mitochondria (mKeima) and a characteristic shift in the absorption/excitation spectrum is observed upon initiation of mitophagy (Figs. 2C and S2C) (Lazarou et al., 2015). We tested each compound in a 7-point concentration curve in the presence of a low concentration (1 μM) of FCCP and oligomycin (FO). In cell culture models, low levels of FO are necessary to trigger mitochondrial stress and stabilize PINK1; this dose was selected because it did not robustly trigger mitophagy on its own.

In order to rule out nonspecific, additive mitochondrial toxicity as the mechanism of action of a compound showing activity in the mKeima assay, we counter-screened active compounds for mitochondrial toxicity in a galactose/glucose cell growth assay (Arroyo et al., 2016; Gohil et al., 2010; Marroquin et al., 2007). We used a 20% decrease in growth rate in galactose-rich media relative to glucose-rich media as a cutoff for mitotoxicity (Figs. 2D and S2A). A subset of these active, non-mitotoxic compounds were then evaluated for initial developability using the following criteria: solubility, permeability, brain efflux, in vitro liver microsome clearance in multiple species, CYP screening, plasma protein binding, and hERG inhibition (Fig. 2). Several compounds that fulfilled developability criteria were tested in mouse pharmacokinetic (PK) and tissue distribution studies. The compound MTK458 showed good potency, no observable mitotoxicity, attractive oral pharmacokinetics (PK), and high brain penetrance. To further confirm that MTK458 is not impairing mitochondria with a more subtle effect, we measured mitochondrial respiration rates in HeLa cells treated with MTK458 for 1 hour. MTK458 did not affect basal respiration, maximal respiration, or spare respiratory capacity (Figs. 2E and S2D).

**Figure 3:**
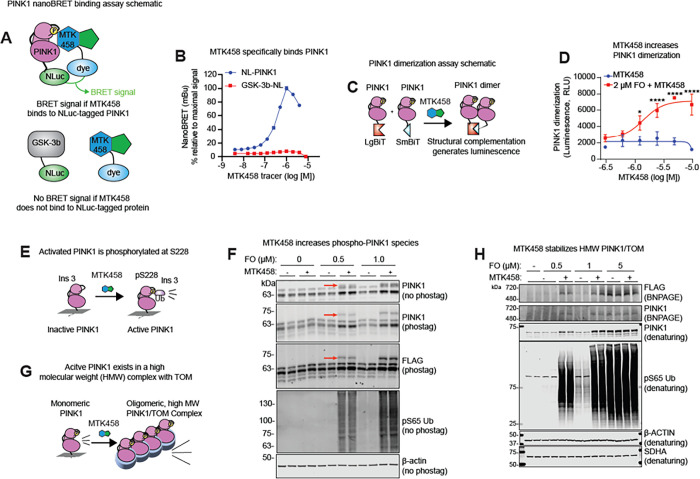
MTK458 stabilizes and sustains the active form of PINK1. (A) Schematic for nanoBRET assay. The nanoBRET assay assesses whether a small molecule (labeled with a BODIPY dye) binds to a particular protein (tagged with NanoLuc). Only when the drug binds to the protein will there be energy transfer between the NanoLuc and the nanoBRET dye, generating a BRET signal. (B) HEK293 PINK1-KO cells were transfected with plasmids encoding PINK1 N-terminally tagged with nanoLuc (NL-PINK1), or GSK-3b C-terminally tagged with NanoLuc (GSK-3b-NL). Cells were then treated with MTK458 labeled with the nanoBRET 590 BODIPY dye (MTK458 tracer). A high nanoBRET signal was observed with NL-PINK1, but not GSK-3b-NL, suggesting specific binding between PINK1 and MTK458. (C) Schematic of PINK1 dimerization (nanoBiT) assay. Plasmids encoding PINK1 tagged with either LgBiT or SmBiT were transfected into YPMK PINK1 KO cells. The dimerization of PINK1-LgBiT and PINK1-SmBiT brings the LgBiT and SmBiT together, forming an active luciferase enzyme capable of generating a luminescent signal. (D) YPMK PINK1 KO cells expressing PINK1-LgBiT and PINK1-SmBiT were treated with 2 μM FO and MTK458, and dimerization (as luminescence) was measured and plotted. (E) Schematic of PINK1 autophosphorylation at S228 generating the active form of PINK1. (F) In EPF1 cells (cells overexpressing PINK1-FLAG) treated with FO and MTK458, there is an increase in the phospho-PINK1 species (red arrow). (G) Schematic of the active, high molecular weight PINK1/TOM complex. (H) EPF1 cells were treated with FO and 2.8 μM MTK458 for 2.5 hours. Whole cell lysates were analyzed by immunoblotting on blue native gels (BNPAGE) or denaturing gels, showing stabilization of the active, high molecular weight PINK1 complex by MTK458. Where applicable, mean and SD are shown and one-way ANOVA was used for statistical analysis. *p < 0.05, **p < 0.01, ***p < 0.001, n.s., not significant.

We tested MTK458 in successive assays for PINK1 pathway activity in the presence of low concentrations of FO. In HeLa cells expressing YFP-Parkin and mito-Keima (YPMK), MTK458 increased pUb as assessed by a custom pUb ELISA assay (Fig. 2F) and mass spectrometry (Figure S2B). Next, we monitored Parkin activation by PINK1 through its cytosolic-to-mitochondrial translocation via live-cell imaging. MTK458 accelerated localization of YFP-Parkin to the mitochondria with a clear dose-responsiveness. (Figs. 2G and S2E). Thus, MTK458 increases early stage (pUb), mid-stage (Parkin recruitment to mitochondria), and late-stage (mitophagy, Fig. 3H) processes of the PINK1/Parkin cascade working in a dose-dependent and PINK1-dependent manner.

PINK1 also phosphorylates Parkin at S65, and downstream of ubiquitin and Parkin phosphorylation, the mitofusin proteins (e.g., MFN1/2) and some outer mitochondrial membrane proteins (e.g., VDAC) are degraded. Consistent with PINK1 activation, MTK458 increases pS65 Parkin and decreases MFN1, MFN2, and VDAC (Figures S3A and S3C-G). PINK1 activation also results in phosphorylation of Rab proteins, specifically Rab8A, 8B and 13, at the highly conserved residue of serine 111 (Lai et al., 2015). The phosphorylation of the Rabs is not catalyzed by PINK1 directly, but is abolished in PINK1 knockout cells, indicating this phosphorylation site can be used as a downstream proxy for PINK1 activity. Consistent with being a PINK1 activator, MTK458 also increases the pS111 Rab8A signal in cells treated with a low dose of FO (Figures S3B and S3H). In the absence of any mitochondrial stressor MTK458 does not induce any of the aforementioned PINK1 biomarkers (Figure S3I), further displaying that the induction and increased activation of mitophagy pathways through MTK458 is selective for dysfunctional states of mitochondria.

### MTK458 shows direct PINK1 binding

Although PINK1 from non-mammalian species has been utilized for structural studies (Gan et al., 2022), purification of human PINK1 for direct binding assays or crystallization has not yet been achieved. In order to demonstrate direct PINK1 engagement we developed a novel direct binding assay for PINK1 in human cells based on the nanoBRET (bioluminescence resonance energy transfer) system (Machleidt et al., 2015), using a tracer molecule based on the structure of MTK458. In this system, PINK1 was N-terminally tagged with NanoLuc luciferase (NL-PINK1), and MTK458 was labeled with the nanoBRET 590 dye. With this approach, a BRET signal only results if the labeled MTK458 is within 100 angstroms of NL-PINK1 (Fig. 3A). We observed a concentration dependent increase in BRET signal in cells expressing NL-PINK1 and treated with the MTK458-derived nanoBRET tracer (Fig. 3B), suggesting that MTK458 binds directly to PINK1. As a control, MTK458 did not bind an unrelated but luciferase-tagged kinase, GSK3 (GSK3B-NL), Fig. 3B). When we used a non-specific kinase binding tracer K8 (Promega) induced a dose-dependent increase in BRET ratio with the GSK3B-NL, and less signal with NL-PINK1 (Figure S4A), suggesting that MTK-458 promotes mitophagy through direct and specific binding of PINK1.

### MTK458 stabilizes the PINK1/TOM complex and opposes PINK1 inactivation

**Figure 4:**
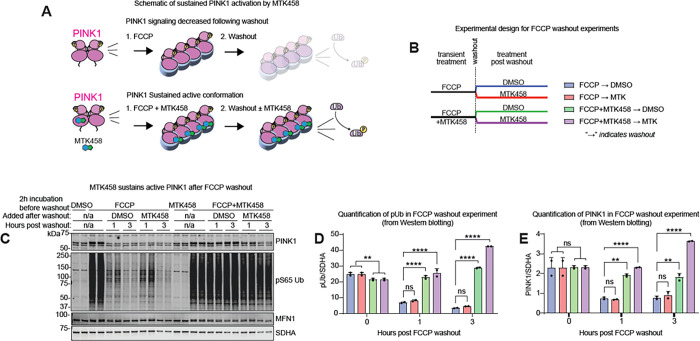
PINK1 activator MTK458 rescues proteotoxicity and PD pathology in immortalized cells, primary neurons, and iPSC-derived neurons. (A) A schematic of PINK1 sustained active conformation with MTK458 treatment is shown. (B) A schematic of the FCCP washout experiment in (C-E). SK-OV-3 cells were treated with 10 μM FCCP or 10 μM FCCP + 2.8 μM MTK458 for 2 hours. Cells were washed 3 times to remove FCCP (“washout”), and then media containing either DMSO or MTK458 was added back to the cells. Cells were harvested for analysis just before the washout, or 1 hour or 3 hours after the washout. (C) Immunoblot analysis of cells collected in the FCCP washout experiment, showing sustained PINK1 stability and activity in cells that experienced co-treatment of FCCP with MTK458. (D-E) Band intensities in (C) were quantified and plotted. Where applicable, mean and SD is shown; one-way ANOVA was used for statistical analysis. *p < 0.05, **p < 0.01, ***p < 0.001, ****p < 0.0001, n.s., not significant.

We next explored the mechanism by which MTK458 potentiates PINK1 activity. Previous work with kinetin and the active metabolite KTP suggested that modification to a triphosphate form is required for activity (Hertz et al., 2013). However, MTK458 cannot be ribosylated (data not shown), so despite similarities in structure, we postulated that MTK458 must act via a new mechanism. Activation of PINK1 is believed to involve dimerization, auto-phosphorylation in trans at Ser228, and formation of a high molecular weight (HMW) complex with components of the mitochondrial translocase of the outer membrane (TOM) proteins (Lazarou et al., 2012; Okatsu et al., 2012; Rasool and Trempe, 2018). To investigate the effect of MTK458 on PINK1 dimerization, we used a split-nanoLuc protein fragment complementation system whereby cells were transfected with two species of PINK1, one fused with SmBiT and the other with LgBiT (Fig. 3C). When PINK1 dimerizes, the SmBiT and LgBiT proteins assemble into a functional nanoLuc protein that can generate a luminescence signal. MTK458 increased PINK1 dimerization in a concentration dependent manner (Fig. 3D). Importantly, MTK458 or low-dose F/O alone (t = 0 point is + F/O) did not stimulate PINK1 dimerization when applied separately, while FO priming in combination with MTK458 treatment resulted in a robust PINK1 dimerization as evidenced by increased luminescence signal in this assay. Next, we used Phos-tag SDS-PAGE (Kinoshita et al., 2009) and blue-native gel electrophoresis (Lazarou et al., 2012) to test the effect of MTK458 on PINK1 phosphorylation and complex formation, respectively. We observed an increase in phospho-PINK1 by MTK458 in Phos-tag SDS-PAGE (Figs. 3E–F and S4B). When lysates from cells exposed to high F/O were treated with lambda protein phosphatase, the intensity of the phospho-PINK1 band and pUb bands decreased (data not shown). Besides phosphorylation, addition of MTK458 increased the total amount of PINK1 in the active, HMW complex (Figs. 4G–H and S4C), suggesting that MTK458 treatment increased the levels of total and phosphorylated PINK1.

In the absence of mitochondrial stress, PINK1 is rapidly destabilized and degraded (Jin et al., 2010). We found that MTK458 does not activate PINK1 without a mitochondrial stressor, whereas it potentiates both PINK1 autophosphorylation and complex formation with low-dose mitochondrial stress. Based on this finding, we hypothesized that MTK458 stabilizes the active PINK1 complex and therefore delays its inactivation. To investigate the effect of MTK458 on PINK1 complex stability after removal of mitochondrial toxins, we performed FCCP washout studies in SK-OV-3 cells, which express endogenous levels of Parkin and downstream components of the PINK1/parkin pathway (Kakade et al., 2022) (Fig. 4A). SK-OV-3 cells were transiently treated with FCCP alone or combined FCCP/MTK458 for 2h, then the FCCP was removed by washing the cells three times with FBS-containing medium (“washout”). After the washout, the cells were treated with eitherMTK458 or DMSO control (Fig. 4B). Exposure to FCCP induced high PINK1 and pUb levels, which rapidly decreased after washout in the DMSO condition (Figs. 4C–E and S4D). However, if the cells were co-treated with MTK458 during the transient FCCP treatment, the high PINK1 and pUb levels were sustained after the washout as detected by both immunoblotting and mass spectrometry (Ordureau et al., 2014) (Figs. 4C–E and S4D). The high molecular weight PINK1 complex was also sustained by MTK458 even after FCCP is removed (Figures S4E-F). Importantly, MTK458 treatment did not interfere with mitochondrial repolarization, suggesting that the PINK1 complex-potentiating effect is not driven by a compound-driven effect on mitochondrial membrane potential (Figures S4G-H). Taken together, our data supports a model where MTK458 potentiates and prolongs the stability of the active PINK1 complex (Fig. 4A), but does not initiate complex stabilization without mitochondrial depolarization.

### MTK458 drives clearance of pathologic α-synuclein in vitro

Alpha-synuclein aggregation induces mitochondrial dysfunction coupled, reduced rates of mitophagy and therefore accumulation of pUb in primary neurons (Fig. 1K). Therefore, we wanted to test whether treatment with MTK458 would increase PINK1 activity in models of proteinopathy induced mitochondrial dysfunction. To test this, we utilized two independent proteinopathy models, an inducible mitochondrial proteinopathy model and the aggregated α-synuclein seeding model (PFFs) noted above.

**Figure 5:**
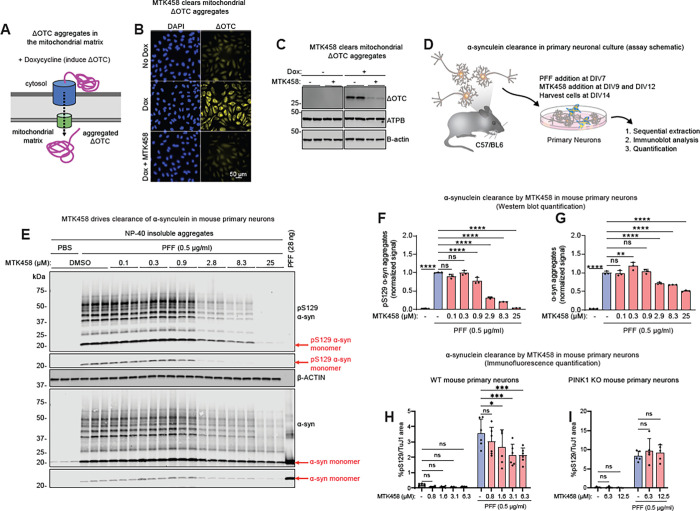
PINK1 activator MTK458 rescues proteotoxicity and PD pathology in immortalized cells, primary neurons, and iPSC-derived neurons. (A) Schematic describing the inducible cell-based model expressing a deletion mutant of ornithine transcarbamylase (ΔOTC). This mutant yields detergent-insoluble protein aggregates in the mitochondrial matrix. (B-C) In HeLa cells expressing YFP-Parkin and doxycycline-induced ΔOTC, MTK458 treatment (25 μM) results in the clearance of ΔOTC, as assessed by immunofluorescence (B) or immunoblotting (C). (D) Schematic describing the primary neuron culture experiments in (E-G). Mouse primary cultured neurons were challenged with PFFs (0.5 ug/mL) on DIV7, treated with MTK458 on DIV9 and DIV12, and then harvested on DIV14. (E) Following serial extraction, the amount of pS129 α-syn in the NP-40 insoluble fraction was quantified from immunoblots, showing a dose dependent decrease in both pS129 α-syn aggregates and α-syn aggregates (12–250 kDa) by MTK458. (F-G) Quantification of (E) is shown. (H-I) WT or PINK1 KO mouse primary cultured neurons were challenged with PFFs and treated with MTK458 as in (D), but pS129 α-syn was analyzed by immunofluorescence. MTK458 dose-dependently decreases pS129 α-syn signal in WT neurons, but not PINK1 KO neurons. Where applicable, mean and SD is shown; one-way ANOVA was used for statistical analysis. *p < 0.05, **p < 0.01, ***p < 0.001, ****p < 0.0001, n.s., not significant.

First, we utilized a cell-based model, which expresses a deletion mutant of ornithine transcarbamylase (ΔOTC) (Fig. 5A) (Moisoi et al., 2014). This mutanyields detergent-insoluble, intra-mitochondrial ΔOTC protein aggregates within the mitochondrial matrix that can be cleared by PINK1/Parkin-mediated mitophagy (Burman et al., 2017; Moisoi et al., 2014). In HeLa cells expressing doxycycline-inducible ΔOTC and YFP-Parkin, enhancing PINK1 activity with MTK458 treatment resulted in the robust clearance of ΔOTC as measured by either immunofluorescence or Western blotting (Figs. 5B–C), demonstrating that clearance of intra-mitochondrial aggregates can be enhanced by increased PINK1-mediated mitophagy driven by MTK458.

Having shown that MTK458 treatment could reduce artificially induced intra-mitochondrial aggregates in non-neuronal cells, we next tested whether PINK1 activation could ameliorate PD-patient relevant pathology in mouse and human neurons in vitro. Primary mouse hippocampal neuron cultures from PINK1wt (5E-H) or PINK1 knockout (KO) (5I) mice seeded with α-synuclein PFFs on DIV7 (Volpicelli-Daley et al., 2014) were allowed to seed and further develop detergent insoluble pS129 α-synuclein aggregates prior to the addition of MTK458 on DIV9 and DIV12 (Fig. 5D). On DIV14, pS129 α-synuclein aggregates were detectable by immunoblotting from the insoluble fraction (Figs. 5E–G) and immunofluorescence (Figs. 5H–I). MTK458 treatment led to the clearance of pS129 α-synuclein aggregates (12–250 kDa) in a dose and PINK1 dependent manner (Figs. 5E–I).

We further tested the effect of MTK458 in iPSC-derived neurons from patients carrying the A53T-α-synuclein mutation associated with familial PD (Figure S5A). This line carries an A53T α-synuclein mutation causing the derived DA neurons to accumulate pS129 α-synuclein without the addition of exogenous PFFs, and additionally serves to bridge primary mouse neuron studies with human neurons. To first test if global induction of mitophagy driven could reduce pS129 α-synuclein, cells were treated with FCCP to activate PINK1 by depolarization (Figures S5B-D). FCCP alone reduced pS129 α-synuclein pathology in these cells, but at the expense of an increase in mitochondrial stress throughout the cell, as evidenced by stabilization of PINK1 and increased pUb levels (Figures S5B-D). In contrast, MTK458 treatment for 10 days reduced α-synuclein pathology and the mitochondrial stress marker pUb (Figures S5B-D). We hypothesize that mitochondrial depolarization is being triggered by a-synuclein pathology in the absence of FCCP and PINK1 stabilization is further induced in cells treated with MTK458, but at lower levels as compared to FCCP treatment and more selectively on impaired mitochondria. Consistent with the model we proposed above, activation of PINK1 in the patient-derived iPSC neurons reduced protein aggregate load and ultimately drove a reduction in pUb (Figures S5B-D), indicative of clearance of damaged mitochondria. Our results in various PD cell models suggest that pharmacological augmentation of PINK1 activity can ameliorate proteinaceous pathology in vitro .

### MTK458 drives clearance of pathologic α-synuclein in vivo

**Figure 6:**
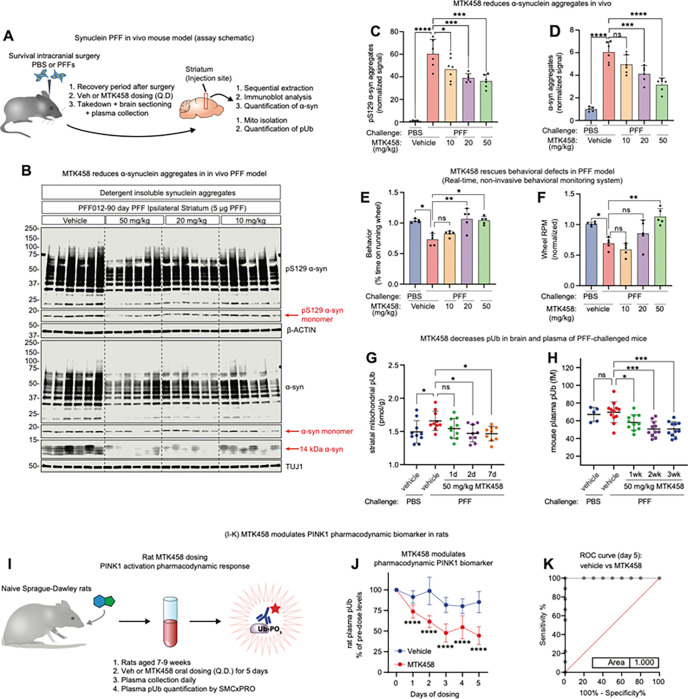
PINK1 activator MTK458 rescues PD pathology and normalizes p-S65-Ub levels in vivo (A) Schematic of *in vivo* PFF experiments in mice. Mice were challenged with striatal injection of PFFs and dosed (QD, PO) with the indicated dose levels of MTK458. (B) After 90 days, striatum brain pieces were analyzed for pS129 α-syn aggregates or total α-syn aggregates (12–250 kDa). (C-D) Quantification of analysis of the full cohort is shown here. (E-F) Parallel mice were challenged with striatal injection of PBS or PFF, and then dosed with MTK458 for 6 months. Mice were analyzed for time spent on the running wheel using the Vium system. MTK458 treatment rescued the reductions in overall wheel activity and wheel speed (RPM) induced by PFF challenge. (G) Mice were challenged with striatal injection of PFFs and after 3 months, were dosed (QD, PO) with 50 mg/kg MTK458 for the indicated times. Mitochondria was isolated from striatal brain pieces and analyzed for pUb content on the MSD assay. (H) Mice were challenged with PFFs, and after 3 months, were dosed (PO, QD) with 50 mg/kg MTK458 for the indicated durations. Plasma pUb levels were measured by the SMCxPRO pUb assay. (I) Schematic of rat dosing study to evaluate plasma pUb as a target engagement biomarker for PINK1 activator compounds. (J) Naïve Sprague-Dawley rats were dosed (PO, QD) with 50 mg/kg MTK458 for 5 days (6 doses). Plasma pUb levels at the indicated timepoints were determined by the SMCxPRO pUb assay. (K) ROC curve for plasma pUb levels in vehicle vs MTK458 dosed rats (after 5 days of dosing) is shown. Where applicable, mean and SD is shown; one-way ANOVA was used for statistical analysis. *p < 0.05, **p < 0.01, ***p < 0.001, ****p < 0.0001 n.s., not significant.

To test if PINK1 activation could rescue α-synuclein pathology in vivo, we utilized a widely adopted pre-clinical model for PD in which α-synuclein PFFs are injected unilaterally into the striatum of mice, leading to progressive spread α-synuclein pathology (Fig. 6A) (Luk et al., 2012). Microdialysis studies with MTK458 in the mouse striatum showed a similar unbound plasma and brain exposures at equilibrium (unbound partition coefficient, Kp _u,u_ ~1) (data not shown) showing that MTK458 has excellent mouse pharmacokinetics and high brain penetrance. Consistent with our finding in mouse primary neuron cultures that PFF injection led to profound α-synuclein pathology in the striatum as evidenced by aggregated and pS129 α-synuclein after 12 weeks of incubation accompanied by an increase in pUb (Figs. 6B–D and 6G). PFF, but not PBS injection, increases brain pUb (Fig. 6G), and central (TREM2) and peripheral (IL6, CXCL1) inflammatory markers (Figures S6A-C). Daily oral administration of MTK458 in these mice led to dose dependent decrease (up to ~ 50%) in α-synuclein pathology in 3-month studies (Figs. 6B–D). MTK458 also rescued an activity deficit in freely moving PFF-seeded mice as assessed by home cage monitoring (Figs. 6E–F) (Lim, M.A., et al 2017 Frontiers in Pharmacology). The dose-response rescue in pathology matched the rescue in motor activity. The increase in TREM2, IL-6, and CXCL1 inflammatory markers were also attenuated by MTK458 (Figures S6A-C), which is in line with a proposed role for PINK1 in inflammation (Sliter et al., 2018).

Consistent with the results from our iPSC-derived neuron experiments, MTK458 treatment decreased pUb in the brains of PFF seeded mice (Fig. 6G), suggesting a reduction in mitochondrial stress level due to the clearance of damaged mitochondria and pS129 α-synuclein aggregates. Unexpectedly, we did not see an increase in plasma pUb levels in the PFF-challenged mice as compared to PBS challenged mice. However, a decrease in plasma pUb was observed in mice treated with MTK458 (Fig. 6H) showing global PINK1 pathway engagement.

Since plasma concentrations of pUb in naïve, wild-type animals are measurable and greater than in PINK1 KO animals (Chin et al., 2023), we hypothesized that we might be able to detect a decrease in pUb after a short-term MTK458 treatment in animals. Such a change would be useful to measure target engagement of PINK1 activator compounds in animals or in patients. To test this hypothesis, we dosed naïve, wild-type Sprague-Dawley rats for five days with either a vehicle control or 50 mg/kg MTK458 and found a significant decrease in pUb compared to the vehicle-treated or pre-dosed rats (Figs. 6I–J). The magnitude of the plasma pUb lowering effect (ROC = 1.00) (Figs. 6K and S6E-H) suggests it may be useful as a specific and sensitive pharmacodynamic biomarker for MTK458 treatment.

## Discussion

In this study, we showed that α-synuclein pathology localizes to mitochondria, causing mitochondrial dysfunction, stalled mitophagy, and increased pUb levels. To address these mitochondrial deficits and more specifically reduced mitophagy, we hypothesized that pharmacological activation of PINK1 could be a valuable strategy to ameliorate these impairments. Therefore, we chose the only known PINK1 activator, kinetin, as a chemistry starting point and synthesized hundreds of drug-like kinetin analogs and tested their ability to activate PINK1. In this process, we discovered the small molecule MTK458, which binds to and stabilizes the active form of PINK1, increasing its activity and activating mitophagy downstream. In both cellular and animal PD models, PINK1 activation with MTK458 alleviated the hallmark α-synuclein aggregation, mitochondrial dysfunction, and stalled mitophagy that occurs in our PD models. PINK1 activation also reduced the PINK1-specific biomarker pUb, an indicator of mitochondrial stress. Our data serves as a preclinical proof-of-concept supporting PINK1 activation as a strategy for addressing pathologies implicated in Parkinson’s disease.

### PINK1 as a target for disease modification

PINK1 has several features that make it inherently attractive as a drug target. First, loss of function mutations in PINK1 are genetically linked to Parkinson’s disease. Second, mitochondrial dysfunction is associated with PD and PINK1 activity plays a central role in triggering stress-related mitochondrial quality control processes, suggesting that PINK1 activation could have a disease-modifying benefit (Borsche et al., 2021; Kim et al., 2021). Third, PINK1 has an endogenous regulatory mechanism that limits its presence only to conditions involving mitochondrial stress, so pharmacological activation of PINK1 should not constitutively activate kinase activity. An alternative target could be the E3-Ubiquitin ligase Parkin, which is also genetically linked to PD. However, Parkin is a less attractive therapeutic target because it is present constitutively, can affect proteasome-mediated degradation, and is difficult to activate pharmacologically (Shlevkov et al., 2022).

Our group and others previously published that the neo-substrate kinetin tri-phosphate (KTP) could be used as an alternative phospho-donor by PINK1 with higher catalytic efficiency than ATP, and that the pro-drug kinetin could be taken up by cells and converted to KTP (Hertz et al., 2013; Osgerby et al., 2017). Others have shown that kinetin can activate PINK1 and rescue the mitochondrial mutation load and climbing activity in heteroplasmic flies in a PINK1-dependent manner (Tsai et al., 2022). However, kinetin is not developable due to low potency, low brain penetration, and unfavorable pharmacokinetics, which limited its efficacy in mammalian in vivo models (Orr et al., 2017). Using kinetin as a starting point, we searched for more potent molecules with attractive drug-like properties following a structure-activity-relationship (SAR)-driven approach. We screened compounds using both mitophagy (efficacy) and mitochondrial toxicity (safety) assays to eliminate potential hits that were simply mitochondrial toxins. We then confirmed that the active compounds resulted in the activation of the entire PINK1 signaling cascade, including Ub phosphorylation, Parkin recruitment, and mitophagy.

We identified MTK458 as a PINK1 activator that directly binds to PINK1 and stabilizes the active form of PINK1. MTK458 retains the mitophagy activating properties of kinetin but has enhanced pharmacokinetics, including brain penetration and improved metabolic and physiochemical properties. Using MTK458 to activate mitophagy restored cellular quality control capabilities for mitochondrial homeostasis and resulted in reduced levels of pathogenic α-synuclein *in vitro* and *in vivo*. Despite being observed by several labs (Butler et al., 2012; Lee et al., 2002; Nakamura et al., 2011; Ryan et al., 2018; Shaltouki et al., 2018), the detailed mechanism of how mitophagy and improved mitochondrial homeostasis lead to mitochondrial protein aggregate clearance is not known. The amelioration of α-synuclein pathology may be a direct result of clearing the mitochondria associated α-synuclein or an indirect result of the increased mitochondrial homeostasis, which would stabilize ATP levels leading to more lysosomal andproteasomal activity for the overall improvement in cellular proteostasis.

### PINK1 substrate pUb biomarker mechanism

**Figure 7:**
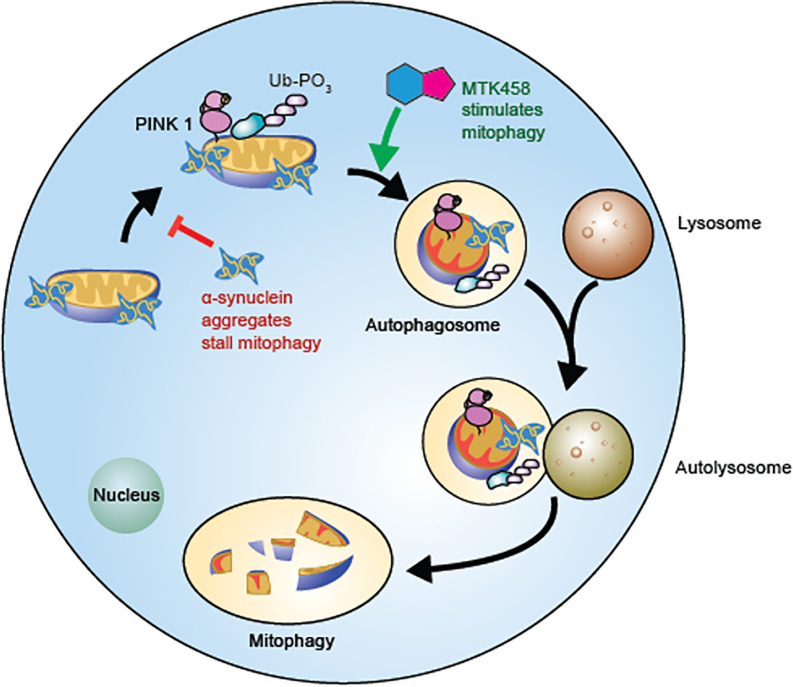
Schematic of pUb regulation. Model for the increased pUb induced by α-synuclein aggregates, which is opposed by MTK458. Cells can dispose of dysfunctional mitochondria by PINK1/Parkin mediated mitophagy. α-synuclein aggregates stall mitophagy and result in an increase of pUb inside the cell. MTK458 increases PINK1/Parkin mediated mitophagy, reducing α-synuclein aggregates and pUb.

Our finding that pUb is elevated in PD models and that a PINK1 activator reduces pUb may seem unexpected. However, it is consistent with several features of our selective, PINK1-targeted strategy and the interplay between α-synuclein pathology and mitochondrial dysfunction. Our model is shown in Fig. 7. Under basal mitochondrial stress, PINK1 is stabilized in depolarized regions of mitochondria, resulting in fragmentation, engulfment of the damaged piece of mitochondrial network by autophagic machinery, and restoration of mitochondrial health. In PD models, PINK1 is activated and pUb is increased due to aggregated α-synuclein at the mitochondria. Normally, pUb spikes would be quickly resolved by mitophagy. However, because mitophagy is compromised by α-synuclein aggregation (Fig. 1J) (Shaltouki et al., 2018), the increase in pUb is stabilized and detectable in cells and tissues (Figs. 1K and 6G). Ultimately, if the pathology remains unresolved, α-synuclein aggregation leads to Lewy body formation and eventual neuronal death. This pathology can be rescued with compounds that activate PINK1, such as MTK458. As we showed in preclinical models of PD, treatment with a PINK1 activator rescued mitophagy, decreased α-synuclein aggregation, and lowered pUb in the brain and plasma (Fig. 7).

## Summary

In conclusion, our data support the idea that increasing PINK1 activity to induce mitophagy could be a viable therapeutic approach for disease-modification in idiopathic PD. Data from our group and others suggest a model whereby α-synuclein pathology causes mitochondrial dysfunction and suppresses mitophagy, thereby increasing pUb. The novel small molecule MTK458 binds to PINK1 and stabilizes its active complex, triggering the first step in mitophagy. In both cellular and animal models of α-synuclein aggregation (PD-like pathology), MTK458 decreased pS129 α-synuclein aggregates and normalized both brain and corresponding plasma pUb levels. PINK1 activation may thus be sufficient to address the hallmark α-synuclein pathology observed in PD and the resultant mitochondrial dysfunction. Altogether, our data demonstrate that PINK1 activators can rescue pathology associated with idiopathic PD and that this class of molecules is worthy of further preclinical and clinical exploration as potential disease modifying therapeutics for PD.

## Data Availability

All data needed to evaluate the conclusions of the paper are present in the paper and/or the supplementary materials.

## Highlights

1. Identification of a potent, brain penetrant PINK1 activator, MTK458
2. MTK458 selectively activates PINK1 by stimulating dimerization and stabilization of the PINK1/TOM complex
3. MTK458 drives clearance of α-synuclein pathology and normalizes pUb in in vivo Parkinson’s models
